# Supplementary material for: Reduction of antimicrobial resistant pneumococci seven years after introduction of pneumococcal vaccine in Iceland
Source: PLoS One. 2020 Mar 17;15(3):e0230332. doi: 10.1371/journal.pone.0230332 (PMC7077842; doi:10.1371/journal.pone.0230332)
Supplement: S1 Table — (DOCX) [file pone.0230332.s001.docx]

Supplementary table 1. Annual numbers and proportions of samples, total number of pneumococcal isolates, penicillin non-susceptible pneumococci (PNSP) and there of vaccine serotypes (VT) and n**on-vaccine serotypes (NVT), all** according to sampling site.

|  | **2011** | **2012** | **2013** | **2014** | **2015** | **2016** | **2017** | **Total** |
| --- | --- | --- | --- | --- | --- | --- | --- | --- |
| **Middle ear** |  |  |  |  |  |  |  |  |
| Samples, n | 950 | 849 | 894 | 687 | 479 | 505 | 504 | 4,868 |
| Pneumococcal isolates, n | 191 | 133 | 146 | 68 | 46 | 47 | 36 | 667 |
| Pneum. isol./samples, % | 20.1 | 15.7 | 16.3 | 9.9 | 9.6 | 9.3 | 7.1 | 88 |
| PNSP isolates. n | 97 | 46 | 34 | 14 | 10 | 11 | 16 | 228 |
| PNSP/samples, % | 10.2 | 5.4 | 3.8 | 2.0 | 2.1 | 2.2 | 3.2 | 4.7 |
| PNSP/pneum. isolates, % | 50.8 | 34.6 | 23.3 | 20.6 | 21.7 | 23.4 | 44.4 | 34.2 |
| PNSP of VT, n | 94 | 36 | 28 | 6 | 1 | 2 | 2 | 169 |
| VT/PNSP, % | 96.9 | 78.3 | 82.4 | 42.9 | 10.0 | 18.2 | 12.5 | 74.1 |
| PNSP of NVT, n | 3 | 10 | 6 | 8 | 9 | 9 | 14 | 59 |
| NVT/PNSP, % | 3.1 | 21.7 | 17.6 | 57.1 | 90.0 | 81.8 | 87.5 | 25.9 |
| **Lower respiratory tract** |  |  |  |  |  |  |  |  |
| Samples, n | 1,676 | 1,750 | 1,671 | 1,620 | 2,176 | 2,384 | 2,356 | 13,633 |
| Pneumococcal isolates, n | 85 | 108 | 86 | 75 | 89 | 91 | 85 | 619 |
| Pneum. isol./samples, % | 5.1 | 6.2 | 5.1 | 4.6 | 4.1 | 3.8 | 3.6 | 4.5 |
| PNSP isolates, n | 29 | 43 | 34 | 25 | 26 | 37 | 30 | 224 |
| PNSP/samples, % | 1.7 | 2.5 | 2.0 | 1.5 | 1.2 | 1.6 | 1.3 | 1.6 |
| PNSP/pneum. isolates, % | 34.1 | 39.8 | 39.5 | 33.3 | 29.2 | 40.7 | 35.3 | 36.2 |
| PNSP of VT, n | 25 | 40 | 26 | 18 | 17 | 14 | 13 | 153 |
| PNSP of VT/PNSP, % | 86.2 | 93.0 | 76.5 | 72.0 | 65.4 | 37.8 | 43.3 | 68.3 |
| PNSP of NVT, n | 4 | 3 | 8 | 7 | 9 | 23 | 17 | 71 |
| PNSP of NVT/PNSP, % | 13.8 | 7.0 | 23.5 | 28.0 | 34.6 | 62.2 | 56.7 | 31.7 |
| **Sterile body fluids** |  |  |  |  |  |  |  |  |
| Samples, n | 8,817 | 8,596 | 9,079 | 9,367 | 10,125 | 10,981 | 11,908 | 68,873 |
| Pneumococcal isolates, n | 31 | 25 | 17 | 24 | 24 | 17 | 24 | 162 |
| Pneum. isol./samples, % | 0.4 | 0.3 | 0.2 | 0.3 | 0.2 | 0.2 | 0.2 | 0.2 |
| PNSP isolates, n | 6 | 1 | 3 | 2 | 4 | 3 | 5 | 24 |
| PNSP/samples, % | 0.07 | 0.01 | 0.03 | 0.02 | 0.04 | 0.03 | 0.04 | 0.03 |
| PNSP/pneum. isolates, % | 19.4 | 4.0 | 17.6 | 8.3 | 16.7 | 17.6 | 20.8 | 14.8 |
| PNSP of VT, n | 3 | 1 | 1 | 0 | 2 | 0 | 1 | 8 |
| PNSP of VT/PNSP, % | 50.0 | 100.0 | 33.3 | 0.0 | 50.0 | 0.0 | 20.0 | 33.3 |
| PNSP of NVT, n | 3 | 0 | 2 | 2 | 2 | 3 | 4 | 16 |
| PNSP of NVT/PNSP, % | 50.0 | 0.0 | 66.7 | 100.0 | 50.0 | 100.0 | 80.0 | 66.7 |
| **Other sampling sites** |  |  |  |  |  |  |  |  |
| Pneumococcal isolates, n | 41 | 56 | 50 | 35 | 31 | 27 | 18 | 258 |
| PNSP isolates, n | 11 | 15 | 2 | 1 | 4 | 4 | 3 | 40 |
| PNSP/pneum. isolates, % | 26.8 | 26.8 | 4.0 | 2.9 | 12.9 | 14.8 | 16.7 | 15.5 |
| PNSP of VT, n | 10 | 14 | 0 | 1 | 3 | 3 | 1 | 32 |
| PNSP of VT/PNSP, % | 90.9 | 93.3 | 0.0 | 100.0 | 75.0 | 75.0 | 33.3 | 80.0 |
| PNSP of NVT, n | 1 | 1 | 2 | 0 | 1 | 1 | 2 | 8 |
| PNSP of NVT/PNSP, % | 9.1 | 6.7 | 100.0 | 0.0 | 25.0 | 25.0 | 66.7 | 20.0 |
| **Total** |  |  |  |  |  |  |  |  |
| Samples, n | 11,443 | 11,195 | 11,644 | 11,674 | 12,780 | 13,870 | 14,768 | 87,374 |
| Pneumococcal isolates, n | 348 | 322 | 299 | 202 | 190 | 182 | 163 | 1,706 |
| Pneum. isol./samples, % | 3.0 | 2.9 | 2.6 | 1.7 | 1.5 | 1.3 | 1.1 | 2.0 |
| PNSP isolates, n | 143 | 105 | 73 | 42 | 44 | 55 | 54 | 516 |
| PNSP/samples, % | 1.2 | 0.9 | 0.6 | 0.4 | 0.3 | 0.4 | 0.4 | 0.6 |
| PNSP/pneum. isolates, % | 41.1 | 32.6 | 24.4 | 20.8 | 23.2 | 30.2 | 33.1 | 30.2 |
| PNSP of VT, n | 132 | 91 | 55 | 25 | 23 | 19 | 17 | 362 |
| PNSP of VT/PNSP, % | 92.3 | 86.7 | 75.3 | 59.5 | 52.3 | 34.5 | 31.5 | 70.2 |
| PNSP of NVT, n | 11 | 14 | 18 | 17 | 21 | 36 | 37 | 154 |
| PNSP of NVT/PNSP, % | 7.7 | 13.3 | 24.7 | 40.5 | 47.7 | 65.5 | 68.5 | 29.8 |
