# Supplementary material for: Reduction of antimicrobial resistant pneumococci seven years after introduction of pneumococcal vaccine in Iceland
Source: PLoS One. 2020 Mar 17;15(3):e0230332. doi: 10.1371/journal.pone.0230332 (PMC7077842; doi:10.1371/journal.pone.0230332)
Supplement: S2 Table — (DOCX) [file pone.0230332.s002.docx]

Supplementary table 2. Annual numbers and proportions of samples, total number of pneumococcal isolates, penicillin non-susceptible pneumococci (PNSP) and there of vaccine serotypes (VT) and non-vaccine serotypes (NVT), all according to

age groups.

|  | **2011** | **2012** | **2013** | **2014** | **2015** | **2016** | **2017** | **Total** |
| --- | --- | --- | --- | --- | --- | --- | --- | --- |
| **0- 1 years** |  |  |  |  |  |  |  |  |
| Pneumococcal isolates. n | 154 | 109 | 108 | 54 | 36 | 33 | 29 | 523 |
| PNSP isolates. n | 88 | 35 | 23 | 10 | 4 | 7 | 12 | 179 |
| PNSP of pneum. isolates. % | 57.1 | 32.1 | 21.3 | 18.5 | 11.1 | 21.2 | 41.4 | 34.2 |
| PNSP of VT. n | 85 | 28 | 17 | 4 | 0 | 1 | 2 | 137 |
| PNSP of VT/ pneum. isolates. % | 55.2 | 25.7 | 15.7 | 7.4 | 0.0 | 3.0 | 6.9 | 26.2 |
| PNSP of VT/PNSP. % | 96.6 | 80.0 | 73.9 | 40.0 | 0.0 | 14.3 | 16.7 | 76.5 |
| PNSP/100,000 inhabitants | 1172.1 | 493.7 | 336.7 | 148.9 | 60.9 | 110.0 | 194.9 |  |
| PNSP of VT/100,000 inhabitants | 1132.1 | 395.0 | 248.9 | 59.5 | 0.0 | 15.7 | 32.5 |  |
| PNSP of NVT. n | 3 | 7 | 6 | 6 | 4 | 6 | 10 | 42 |
| PNSP of NVT/ pneum. isolates. % | 1.9 | 6.4 | 5.6 | 11.1 | 11.1 | 18.2 | 34.5 | 8.0 |
| PNSP of NVT/PNSP. % | 3.4 | 20.0 | 26.1 | 60.0 | 100.0 | 85.7 | 83.3 | 23.5 |
| **2-6 years** |  |  |  |  |  |  |  |  |
| Pneumococcal isolates. n | 58 | 46 | 67 | 32 | 22 | 28 | 18 | 271 |
| PNSP isolates. n | 14 | 13 | 12 | 4 | 8 | 6 | 8 | 65 |
| PNSP of pneum. Isolates. % | 24.1 | 28.3 | 17.9 | 12.5 | 36.4 | 21.4 | 44.4 | 24.0 |
| PNSP of VT. n | 14 | 10 | 11 | 2 | 1 | 1 | 1 | 40 |
| PNSP of VT/ pneum. isolates. % | 24.1 | 21.7 | 16.4 | 6.3 | 4.5 | 3.6 | 5.6 | 14.8 |
| PNSP of VT/PNSP. % | 100.0 | 76.9 | 91.7 | 50.0 | 12.5 | 16.7 | 12.5 | 61.5 |
| PNSP/100,000 inhabitants | 83.8 | 75.7 | 68.3 | 22.4 | 45.0 | 34.5 | 47.0 |  |
| PNSP of VT/100,000 inhabitants | 83.8 | 58.3 | 62.6 | 11.2 | 5.6 | 5.8 | 5.9 |  |
| PNSP of NVT. n | 0 | 3 | 1 | 2 | 7 | 5 | 7 | 25 |
| PNSP of NVT/ pneum. isolates. % | 0.0 | 6.5 | 1.5 | 6.3 | 31.8 | 17.9 | 38.9 | 9.2 |
| PNSP of NVT/PNSP. % | 0.0 | 23.1 | 8.3 | 50.0 | 87.5 | 83.3 | 87.5 | 38.5 |
| **7- 17 years** |  |  |  |  |  |  |  |  |
| Pneumococcal isolates. n | 9 | 6 | 5 | 5 | 9 | 1 | 3 | 38 |
| PNSP isolates. n | 0 | 2 | 0 | 0 | 0 | 0 | 0 | 2 |
| PNSP of pneum. Isolates. % | 0.0 | 1.9 | 0.0 | 0.0 | 0.0 | 0.0 | 0.0 | 0.4 |
| PNSP of VT. n | 0 | 1 | 0 | 0 | 0 | 0 | 0 | 1 |
| PNSP of VT/ pneum. isolates. % | 0.0 | 16.7 | 0.0 | 0.0 | 0.0 | 0.0 | 0.0 | 2.6 |
| PNSP of VT/PNSP. % | 0.0 | 50.0 | 0.0 | 0.0 | 0.0 | 0.0 | 0.0 | 50.0 |
| PNSP/100,000 inhabitants | 0.0 | 5.9 | 0.0 | 0.0 | 0.0 | 0.0 | 0.0 |  |
| PNSP of VT/100,000 inhabitants | 0.0 | 2.9 | 0.0 | 0.0 | 0.0 | 0.0 | 0.0 |  |
| PNSP of NVT. n | 0 | 1 | 0 | 0 | 0 | 0 | 0 | 1 |
| PNSP of NVT/ pneum. isolates. % | 0.0 | 16.7 | 0.0 | 0.0 | 0.0 | 0.0 | 0.0 | 2.6 |
| PNSP of NVT/PNSP. % | 0.0 | 50.0 | 0.0 | 0.0 | 0.0 | 0.0 | 0.0 | 50.0 |
| **18-64 years** |  |  |  |  |  |  |  |  |
| Pneumococcal isolates. n | 71 | 96 | 62 | 49 | 46 | 81 | 76 | 481 |
| PNSP isolates. n | 22 | 35 | 22 | 5 | 16 | 26 | 22 | 148 |
| PNSP of pneum. isolates % | 31.0 | 33.3 | 30.1 | 11.9 | 36.4 | 47.3 | 40.7 | 28.7 |
| PNSP of VT. n | 19 | 32 | 14 | 3 | 10 | 11 | 8 | 97 |
| PNSP of VT/ pneum. isolates. % | 26.8 | 33.3 | 22.6 | 6.1 | 21.7 | 13.6 | 10.5 | 20.2 |
| PNSP of VT/PNSP. % | 86.4 | 91.4 | 63.6 | 60.0 | 62.5 | 42.3 | 36.4 | 65.5 |
| PNSP/100,000 inhabitants | 14.1 | 23.8 | 14.9 | 3.3 | 10.5 | 16.9 | 14.0 |  |
| PNSP of VT/100,000 inhabitants | 12.1 | 21.8 | 9.5 | 2.0 | 6.6 | 7.2 | 5.1 |  |
| PNSP of NVT. n | 3 | 3 | 8 | 2 | 6 | 15 | 14 | 51 |
| PNSP of NVT/ pneum. isolates. % | 4.2 | 3.1 | 12.9 | 4.1 | 13.0 | 18.5 | 18.4 | 10.6 |
| PNSP of NVT/PNSP. % | 13.6 | 8.6 | 36.4 | 40.0 | 37.5 | 57.7 | 63.6 | 34.5 |
| **≥65 years** |  |  |  |  |  |  |  |  |
| Pneumococcal isolates. n | 56 | 65 | 57 | 62 | 77 | 39 | 37 | 393 |
| PNSP isolates. n | 19 | 20 | 16 | 23 | 16 | 16 | 12 | 122 |
| PNSP of pneum. Isolates. % | 33.9 | 19.0 | 21.9 | 54.8 | 36.4 | 29.1 | 22.2 | 23.6 |
| PNSP of VT. n | 14 | 20 | 13 | 16 | 12 | 6 | 6 | 87 |
| PNSP of VT/ pneum. isolates. % | 25.0 | 30.8 | 22.8 | 25.8 | 15.6 | 15.4 | 16.2 | 22.1 |
| PNSP of VT/PNSP. % | 73.7 | 100.0 | 81.3 | 69.6 | 75.0 | 37.5 | 50.0 | 71.3 |
| PNSP/100,000 inhabitants | 69.6 | 71.1 | 54.9 | 76.2 | 51.1 | 49.3 | 35.8 |  |
| PNSP of VT/100,000 inhabitants | 51.3 | 71.1 | 44.6 | 53.0 | 38.3 | 18.5 | 17.9 |  |
| PNSP of NVT. n | 5 | 0 | 3 | 7 | 4 | 10 | 6 | 35 |
| PNSP of NVT/ pneum. isolates. % | 8.9 | 0.0 | 5.3 | 11.3 | 5.2 | 25.6 | 16.2 | 8.9 |
| PNSP of NVT/PNSP. % | 26.3 | 0.0 | 18.8 | 30.4 | 25.0 | 62.5 | 50.0 | 28.7 |
