# Supplementary material for: Reduction of antimicrobial resistant pneumococci seven years after introduction of pneumococcal vaccine in Iceland
Source: PLoS One. 2020 Mar 17;15(3):e0230332. doi: 10.1371/journal.pone.0230332 (PMC7077842; doi:10.1371/journal.pone.0230332)
Supplement: S3 Table — (DOCX) [file pone.0230332.s003.docx]

Supplementary table 3. Annual distribution of pneumococcal serotypes and non-encapsulated *Streptococcus pneumonia*(NESp) defined as penicillin non-susceptible pneumococci (PNSP) in 2011-2017, number of isolates (n) and proportions (%).

|  | **2011** | **2012** | **2013** | **2014** | **2015** | **2016** | **2017** | **Total** |
| --- | --- | --- | --- | --- | --- | --- | --- | --- |
|  | n  (%) | n  (%) | n  (%) | n  (%) | n  (%) | n  (%) | n  (%) | n  (%) |
| 19F | 124  (86.7) | 77  (73.3) | 48  (65.8) | 21 (50.0) | 19 (43.2) | 13  (23.6) | 15  (27.8) | 317  (61.4) |
| 6C | 1  (0.7) | 3  (2.9) | 5  (6.8) | 5  (11.9) | 4  (9.1) | 6  (10.9) | 10  (18.5) | 34  (6.6) |
| NESp | 2  (1.4) | 3  (2.9) | 5  (6.8) | 5  (11.9) | 7  (15.9) | 8  (14.5) | 3  (5.6) | 33  (6.4) |
| 6B | 4  (2.8) | 10  (9.5) | 6  (8.2) | 0  (0.0) | 3  (6.8) | 2  (3.6) | 1  (1.9) | 26  (5.0) |
| 15A | 0 (0.0) | 0  (0.0) | 3  (4.1) | 2  (4.8) | 0  (0.0) | 2  (3.6) | 10 (18.5) | 17  (3.3) |
| 19A | 1  (0.7) | 4  (3.8) | 2  (2.7) | 1  (2.4) | 2  (4.5) | 3  (5.5) | 2  (3.7) | 15  (2.9) |
| 14 | 2  (1.4) | 1  (1.0) | 1  (1.4) | 2  (4.8) | 1  (2.3) | 4  (7.3) | 1  (1.9) | 12  (2.3) |
| 23B | 1  (0.7) | 0  (0.0) | 0  (0.0) | 1  (2.4) | 3  (6.8) | 3  (5.5) | 2  (3.7) | 10  (1.9) |
| 35B | 0  (0.0) | 1  (1.0) | 0  (0.0) | 0  (0.0) | 1 (2.3) | 7  (12.7) | 3  (5.6) | 12 (2.3) |
| 15B/C | 0 (0.0) | 1 (1.0) | 0 (0.0) | 0 (0.0) | 1 (2.3) | 2 (3.6) | 0 (0.0) | 4 (1.2) |
| 23F | 1  (0.7) | 3  (2.9) | 0  (0.0) | 2  (4.8) | 0  (0.0) | 0  (0.0) | 0  (0.0) | 6  (1.2) |
| 6A | 2  (1.4) | 1  (1.0) | 1  (1.4) | 0  (0.0) | 1  (2.3) | 0  (0.0) | 1  (1.9) | 6  (1.2) |
| 23A | 1 (0.7) | 0 (0.0) | 0 (0.0) | 0 (0.0) | 1  (2.3) | 0 (0.0) | 3 (5.5) | 5 (1.0) |
| 9A | 1 (0.7) | 0 (0.0) | 1 (1.4) | 1 (2.4) | 0 (0.0) | 0 (0.0) | 0 (0.0) | 3 (0.6) |
| 11A | 0 (0.0) | 0 (0.0) | 0 (0.0) | 0 (0.0) | 1 (2.3) | 1 (1.8) | 0 (0.0) | 2 (0.4) |
| 21 | 0 (0.0) | 1 (1.0) | 0 (0.0) | 1 (2.4) | 0 (0.0) | 0 (0.0) | 0 (0.0) | 2 (0.4) |
| 22F | 0 (0.0) | 0 (0.0) | 0 (0.0) | 0 (0.0) | 0 (0.0) | 2 (3.6) | 0 (0.0) | 2 (0.4) |
| 9V | 1 (0.7) | 0 (0.0) | 0 (0.0) | 0 (0.0) | 0 (0.0) | 0 (0.0) | 0 (0.0) | 1 (0.2) |
| 16F | 0 (0.0) | 0 (0.0) | 1 (1.4) | 0 (0.0) | 0 (0.0) | 0 (0.0) | 0 (0.0) | 1 (0.2) |
| 35F | 0 (0.0) | 0 (0.0) | 0 (0.0) | 1 (2.4) | 0 (0.0) | 0 (0.0) | 0 (0.0) | 1 (0.2) |
| 38 | 1 (0.7) | 0 (0.0) | 0 (0.0) | 0 (0.0) | 0 (0.0) | 0 (0.0) | 0 (0.0) | 1 (0.2) |
| Negative mPCR^a)^ | 1 (0.7) | 0 (0.0) | 0 (0.0) | 0 (0.0) | 0 (0.0) | 2 (3.6) | 3 (5.5) | 6 (1.2) |

^a)^ Included in the PCR panel are serotypes: 1, 3, 4, 5, 6A, 6B, 6C, 6D, 7F, 8, 9V, 9N, 10A, 10F, 11A, 12F, 14, 15A, 15B/C, 16F, 17F, 18A/B/C/F, 19A, 19B/C, 19F, 20A/B, 21, 22F, 23A, 23B, 23F, 24F, 29, 31, 33F,33B/D, 34,35B, 35F, 35(25F), 42 (35A/C), 47A. The isolates defined as negative mPCR were positive for *lyt*A, *cps*A and *cps*B but the serotypes included in the mPCR panel were not detected.
