# Supplementary material for: Reduction of antimicrobial resistant pneumococci seven years after introduction of pneumococcal vaccine in Iceland
Source: PLoS One. 2020 Mar 17;15(3):e0230332. doi: 10.1371/journal.pone.0230332 (PMC7077842; doi:10.1371/journal.pone.0230332)
Supplement: Attachment — Submitted filename: Hjalmarsdottir_Response to reviwers.docx [file pone.0230332.s004.docx]

**Response from the authors to the reviewers**

**Journal Requirements**

1. *When submitting your revision, we need you to address these additional requirements.
   Please ensure that your manuscript meets PLOS ONE's style requirements, including those for file naming. The PLOS ONE style templates can be found at*
   [*http://www.journals.plos.org/plosone/s/file?id=wjVg/PLOSOne_formatting_sample_main_body.pdf*](http://www.journals.plos.org/plosone/s/file?id=wjVg/PLOSOne_formatting_sample_main_body.pdf) *and* [*http://www.journals.plos.org/plosone/s/file?id=ba62/PLOSOne_formatting_sample_title_authors_affiliations.pdf*](http://www.journals.plos.org/plosone/s/file?id=ba62/PLOSOne_formatting_sample_title_authors_affiliations.pdf)

Response

The manuscript has been formatted according to PLOS ONE´s style requirements.
The names of institutions have been amended according to PLOS requirements as follows:

Faculty of Medicine, University of Iceland, Reykjavík, Iceland
Department of Clinical Microbiology, Landspitali University Hospital, Reykjavík, Iceland
BioMedical Centre of the University of Iceland, Reykjavik, Iceland
Children´s Hospital Iceland, Landspitali University Hospital, Reykjavík, Iceland

2. *Please include captions for your Supporting Information files at the end of your manuscript, and update any in-text citations to match accordingly. Please see our Supporting Information guidelines for more information:* [*http://journals.plos.org/plosone/s/supporting-information*](http://journals.plos.org/plosone/s/supporting-information)*.*

Response

Captions for our supporting information have been included at the end of the manuscript as follows:

S1 Table. Annual numbers and proportions of samples, total number of pneumococcal isolates, penicillin non-susceptible pneumococci (PNSP) and there of vaccine serotypes (VT) and non-vaccine serotypes (NVT), all according to sampling site.

S2 Table. Annual numbers and proportions of samples, total number of pneumococcal isolates, penicillin non-susceptible pneumococci (PNSP) and there of vaccine serotypes (VT) and non-vaccine serotypes (NVT), all according to age groups.

S3 Table. The ten most common serotypes of penicillin non-susceptible pneumococci (PNSP) in 2011-2017 and non-encapsulated (NESp) PNSP. Other are the remaining 16 serotypes that were detected in ≥5 isolates each during the study period. All of these are non-vaccine serotypes except for one isolate of 9V in 2011.

3. *In ethics statement in the manuscript and in the online submission form, please provide additional information about the patient records/samples used in your retrospective study. Specifically, please ensure that you have discussed whether all data/samples were fully anonymized before you accessed them and/or whether the IRB or ethics committee waived the requirement for informed consent. If patients provided informed written consent to have data/samples from their medical records used in research, please include this information.*

Response

Additional information about samples and ethics was included as follows:

The samples were bacterial isolates and all patient data was fully anonymized before being were analysed. The National Bioethics Committee waived the requirement for informed consent.

4. *Your ethics statement must appear in the Methods section of your manuscript. If your ethics statement is written in any section besides the Methods, please move it to the Methods section and delete it from any other section. Please also ensure that your ethics statement is included in your manuscript, as the ethics section of your online submission will not be published alongside your manuscript.*

Response

The ethics statement has been written as separate sub chapter in the Material section as follows:

**Ethics statement**
The study was approved by The National Bioethics Committee (VSNb2013010015/03.07) and the appropriate authorities at the Landspitali University Hospital, Iceland. The samples were bacterial isolates and all patient data was fully anonymized before being analysed. The National Bioethics Committee waived the requirement for informed consent.

5. *Please amend your manuscript to include your abstract after the title page.*

Response
The abstract has been included directly after the title page.

6. Thank you for stating the following in the Competing Interests section:

   This work is an investigator-initiated study that was supported by grants from: - The Landspítali University Hospital Research Fund (MÁH) <https://www.landspitali.is>
    - GlaxoSmithKline Biologicals SA (KGK, HE, ÁH), <https://www.gsk.com>
   The funders had no role in study design, data collection and analysis, decision to publish, or preparation of the manuscript.

   We note that you received funding from a commercial source: GlaxoSmithKline Biologicals SA

   Please provide an amended Competing Interests Statement that explicitly states this commercial funder, along with any other [relevant declarations](http://www.plosone.org/static/competing.action) relating to employment, consultancy, patents, products in development, marketed products, etc.

Within this Competing Interests Statement, please confirm that this does not alter your adherence to all PLOS ONE policies on sharing data and materials by including the following statement: "This does not alter our adherence to PLOS ONE policies on sharing data and materials.” (as detailed online in our guide for authors

Response

The Competing Interest Section now reads as follows:

This work is an investigator-initiated study that was supported by grants from: - The Landspítali University Hospital Research Fund (MÁH) <https://www.landspitali.is>
 - GlaxoSmithKline Biologicals SA <https://www.gsk.com>
The authors declare that this does not alter our adherence to PLOS ONE policies on sharing data and materials. Neither GlaxoSmithKline Biologicals SA, nor Landspítali University Hospital Research Fund had any role in study design, data collection and analysis, decision to publish, or preparation of the manuscript.

**Response to reviewer #1**

1. *Materials and Methods: the authors should explain the method of identification of S. pneumoniae in cultures.*

   Response

We agree and added text after the first sentence in the chapter Bacterial isolates and samples as follows (line 99 in manuscript with track changes):

The pneumococci were cultured and identified from routine patient specimens using conventional methods, i.e. plated on two 5% horse blood agar plates (Oxoid, Hampshare, UK), one incubated in a 5% CO_2_ and the other one anaerobically. Identification was done using optochin test (and bile solubility if unclear). All isolates from 2016-2017 were also identified with MALDI-Tof.

2. *Materials and Methods, line 89: “serotyping was done using agglutination with Pneumotest-Latex and/or latex antisera for specified serotypes or pools and/or PCR” – why? Which serotypes were detected by each of the methods?*

Response

We agree that this needs clarification and amended Material and Methods as follows (line 122 in manuscript with track changes):

Oxacillin resistant isolates were routinely screened for vaccine serotypes using agglutination with Pneumotest-Latex and/or latex antisera for specified serotypes or pools (Statens Serum Institute, Copenhagen). Isolates that did not belong to vaccine serotypes were serotyped using PCR.

3. *Materials and Methods, line 97: isolates defined as non-encapsulated when positive for lytA and negative for capsular genes. There are other streptococcal species carrying the lytA gene. Did the authors confirm by any other method the identification of these isolates? Furthermore, non-encapsulated isolates ranked third among NVT(n=34), so this should be clarified.*

   Response
   During the last two years of the study, MALDI-Tof was used in addition to optochin testing for species identification. In an earlier study of our group (Quirk 2019), a large proportion of the isolates were analysed using whole genome sequencing, confirming the identification and the absence of the capsule.

   Following information was added to the results (line 269 in manuscript with track changes):

All the NEsp isolates originated from adults except one from a 12 years old child, 30 of the isolates were from the LRT and three from conjunctiva.

Following text was added to the discussions directly after discussion of NVTs (line 324 in manuscript with track changes):

Interestingly NESp were not found in children while they were among the most common pneumococci in adults. This is in contrast to findings of other investigators who have reported an increase in children after initiation of vaccination (Takeuchi 2019, Keller 2016). In an earlier study of our group a large proportion of the isolates were analysed using whole genome sequencing, confirming the identification and the absence of the capsule (Quirk 2019).

4. *Results, line 153: the number of PNSP changed from 41.1% in 2011 to 20.8% in 2014, but then increased again to 33.1% in 2017. Is the whole period, is really there a decreasing trend? As this is the main message of the paper, I believe it should be further discussed. Furthermore, the dynamics of the serotypes can add extra discussion to this, since the 19F isolates, the main serotype contributing to resistance, decreased from 2011 to 2016, but then increased again in 2017.*

   Response
   The increase in PNSP during the last years of the study are due to increase in NVTs, mainly due to an increase in prevalence of 6C and 15A. The dominating serotype in 2011 was 19F that constantly decreased to 13 isolates in 2016 and their number was 15 in 2017. Of the isolates in 2016 and 2017, two isolates originated from children each year all the other were from adults. We do not know the vaccine status of the children and no change in vaccination recommendations for adults was implemented where the serotype still lingered. We cannot speculate on this low increase of 19F in 2017 as their numbers are so low in 2016 and 2017, but we agree that this needs clarification.

Following sentences sentencewereadded in the discussion (line 290 in manuscript with track changes):

Isolates of serotype 19F were still the majority of PNSP of VT, although only a fraction of their number in 2011, all originating from adults except one. Isolates of other VTs in 2017 were one isolate each of serotype 6B and 14.

5. *Analysis of figure 1 seems to suggest that vaccination was more effective in decreasing the proportion of middle ear infections than that of LRT infections. Can the authors please comment on this?*

Response
The difference can be explained by the difference in the age of the patients. Otitis media is much more common in children. Accordingly, most of the ME samples originate from children. On the other hand, it is difficult to obtain samples from the LRT of small children and pulmonary infection is relatively common in the oldest patients. Accordingly, most of the lower respiratory tract samples originate from adults. A herd effect from the vaccination of children can explain the reduction that is later seen in LRT isolates.

**Reviewer #2**

1. *Abstract. Last paragraph. “linage” should be “lineage”*

   Response
   The authors thank for this comment and have made appropriate correction.
2. *Line 48-57. The paper describes changes in PNSP as percentages of cases throughout the paper. You include population denominators in your methods section. Your population sizes are similar for 2011 and 2017 but showing annual incidence (number of cases/100,000 population) would be a better comparison for showing differences over time*

   Response

The authors agree and thank for this comment. We have added the number of isolates of PNSP/100,000 inhabitants and the number of isolates of VTs of PNSP/100,000 inhabitants to supplementary table 2 and added this information into the results as appropriate (lines 223-228 and 26-238 in manuscript with track changes):

3. *Line 72. Interesting that you included pleural fluid in the LRT group. Why not as sterile body fluid?*

   Response

We agree that pleural fluid is often included in sterile body fluids, however we have considered these LRT samples in our other studies and decided to do the same here.

4. *Line 74. Isolates from “sinuses”? You excluded nasopharynx and throat but kept in sinuses? Why?*

   Response

The authors excluded samples from nasopharynx for two reasons. The first being that during the study period, recommendations to physicians to send nasopharyngeal swabs changed. In the first years no specific recommendations were set, while in the later years the recommendation to physicians were to only send nasopharyngeal swabs from patients with treatment failure after using recommended antimicrobial prescriptions, or from patients with previously known antimicrobial resistant pneumococcal infection. The second reason is that samples from nasopharynx do not distinguish between carrier state and infection. On the other hand, samples from sinuses are mainly taken by otorhinolaryngologists in suspected infection (sinusitis).

5. *Line 159. I assume >0.6-0.5 mg/L should be >0.06-0.5 mg/L?*
   *Fig 3, Please correct your lower values from 0.6 to 0.06 in the legend as well as in the figure.*

Response

The authors thank for this comment and have made appropriate corrections.
