# Supplementary material for: Reduction of antimicrobial resistant pneumococci seven years after introduction of pneumococcal vaccine in Iceland
Source: PLoS One. 2020 Mar 17;15(3):e0230332. doi: 10.1371/journal.pone.0230332 (PMC7077842; doi:10.1371/journal.pone.0230332)
Supplement: Attachment — Submitted filename: Response 2 to reviewers.docx [file pone.0230332.s005.docx]

**Response to reviewer #1**

*In the response to the reviewers, the authors agreed that it was important to discriminate which serotypes were detected by each of the methods. In the revised version, the authors stated that “Isolates that did not belong to vaccine serotypes were serotyped using PCR. The PCR was done as sPCR, or mPCR including a panel of all the serotypes included in the PHiD-CV and selected serotypes previously detected in our studies” – this is contradictory and needs further clarification.

It is clear that the authors identified vaccine serotypes, but among the others, only some were detected, while others were simply classified as NVT. I believe it would be important to know which NVT can the authors identify by the methods used, since one of the main messages of the paper is “NVT in the last years of the study is a cause of concern”. For example, serotype 8 is a NVT emerging in adult IPD in some countries, and the authors never mentioned this serotype. Is it absent from the collection or is simply not included in the serotyping scheme? Additionally, serotypes 35B and 11A have been associated with PNSP, but seem to be also absent from your collection, but they could also be not included in the scheme. I believe this should be clarified, because it is important to know which NVT are increasing.*

Response

The authors misunderstood the reviewer’s comment. We realise this now and agree that the article will benefit from including information on the results from serotyping of all PNSP isolates. For that purpose, we have amended Supplementary table 3 and it now includes these results. Furthermore, we have added a list of all serotypes included in the mPCR panel to the chapter material and methods in lines 124-127 of the manuscript as follows:

The PCR was done as sPCR, or mPCR including a panel of all the serotypes included in the PHiD-CV and selected serotypes previously detected in our studies - serotypes 1, 3, 4, 5, 6A, 6B, 6C, 6D, 6E, 7F, 8, 9V, 9N, 10A, 10F, 11A, 12F, 14, 15A, 15B/C, 16F, 17F, 18A/B/C/F, 19A, 19B/C, 19F, 20A/B, 21, 22F, 23A, 23B, 23F, 24F, 29, 31, 33F,33B/D, 34,35B, 35F, 35(25F), 42 (35A/C), 47A [8, 9, 24-26].

Six isolates were positive in *lyt*A, *cps*A and *cps*B PCRs but negative in the serotype specific PCRs, this is also clarified in the table. Four isolatets of serogroup 15 and two isolates positive to pool G were further serotyped using PCR. The isolates of serogroup 15 were of serotype 15A and the isolates belonging to pool G were of serotype 35B. This was amended in the table and in the manuscript where lines 265-267 now read as follows:

The second most common NVT was serotype 15A with 17/516 (3.3%) isolates, increasing with time. All the 15A isolates were multi-resistant and had similar antibiograms as the serotype 6C isolates. The third was serotype 19A with 15/516 (2.9%) isolates, 1-4 isolates per year over the study period.

Regarding the reviewer’s comment on serotype 8, it is included in the mPCR panel but no isolate of serotype 8 was detected. Only two isolates of serotype 11A were detected, but serotype 35B ranks number ten of PNSP serotypes.

The title of supplementary table 3 has been amended as follows:

Annual distribution of pneumococcal serotypes and non-encapsulated *Streptococcus pneumonia* (NESp) defined as penicillin non-susceptible pneumococci (PNSP) in 2011-2017, number of isolates (n) and proportions (%).
